# Counting Linear Extensions: Parameterizations by Treewidth

**DOI:** 10.1007/s00453-018-0496-4

**Published:** 2018-09-04

**Authors:** E. Eiben, R. Ganian, K. Kangas, S. Ordyniak

**Affiliations:** 1 Department of Informatics, University of Bergen, Bergen, Norway; 2 Algorithms and Complexity Group, TU Wien, Vienna, Austria; 3 Helsinki Institute for Information Technology, University of Helsinki, Helsinki, Finland; 4 Department of Computer Science, University of Sheffield, Sheffield, UK

**Keywords:** Partially ordered sets, Linear extensions, Parameterized complexity, Structural parameters, Treewidth

## Abstract

We consider the $\#\hbox {P}$-complete problem of counting the number of linear extensions of a poset $(\textsc {\#LE})$; a fundamental problem in order theory with applications in a variety of distinct areas. In particular, we study the complexity of $\textsc {\#LE}$ parameterized by the well-known decompositional parameter treewidth for two natural graphical representations of the input poset, i.e., the cover and the incomparability graph. Our main result shows that $\textsc {\#LE}$ is fixed-parameter intractable parameterized by the treewidth of the cover graph. This resolves an open problem recently posed in the Dagstuhl seminar on Exact Algorithms. On the positive side we show that ${\textsc {\#LE}}$ becomes fixed-parameter tractable parameterized by the treewidth of the incomparability graph.

## Introduction

Counting the number of linear extensions of a poset is a fundamental problem of order theory that has applications in a variety of distinct areas such as sorting [30], sequence analysis [25], convex rank tests [27], sampling schemes of Bayesian networks [28], and preference reasoning [24]. Determining the exact number of linear extensions of a given poset is known to be #P-complete [6] already for posets of height at least 3. Informally, #P-complete problems are as hard as counting the number of accepting paths of any nondeterministic Turing machine, implying that such problems are not tractable unless P = NP. The currently fastest known method for counting linear extensions of a general *n*-element poset is by dynamic programming over the lattice of downsets and runs in time $\mathcal {O}(2^n \cdot n)$ [10]. Polynomial time algorithms have been found for various special cases such as series-parallel posets [26] and posets whose cover graph is a (poly)tree [2]. Fully polynomial time randomized approximation schemes are known for estimating the number of linear extensions [7, 13].

Due to the inherent difficulty of the problem, it is natural to study whether it can be solved efficiently by exploiting the structure of the input poset. In this respect, the parameterized complexity framework [9, 12] allows a refined view of the interactions between various forms of structure in the input and the running time of algorithms. The idea of the framework is to measure the complexity of problems not only in terms of input sizes, but also with respect to an additional numerical parameter. The goal is then to develop so-called *fpt algorithms*, which are algorithms that run in time $f(k)n^{\mathcal {O}(1)}$ where *n* is the input size and *f* is a computable function depending only on the parameter *k*. A less favorable outcome is a so-called XP algorithm, which runs in time $n^{f(k)}$; the existence of such algorithms then gives rise to the respective complexity classes ${\textsf {FPT}}$ (*fixed-parameter tractable*) and XP.

The first steps in this general direction have been taken, e.g., in [19], using the decomposition diameter as a parameter, in [15] using a parameter called activity for N-free posets, and very recently in [22], where the treewidth of the so-called cover graph was considered as a parameter. Also the exact dynamic programming algorithm [10] can be shown to run in time $\mathcal {O}(n^w\cdot w)$ for a poset with *n* elements and width *w* (the size of the largest anti-chain). Interestingly, none of these efforts has so far led to an fpt algorithm.

We believe that this uncertainty about the exact complexity status of counting linear extensions with respect to these various parameterizations is at least partly due to the fact that we deal with a counting problem whose decision version is trivial, i.e., every poset has at least one linear extension. This fact makes it considerably harder to show that the problem is fixed-parameter intractable; in particular, the usual approach based on parsimonious reductions fails. On the other hand, the same predicament makes studying the complexity of counting linear extensions significantly more interesting, as noted also by Flum and Grohe [16]:

The theory gets interesting with those counting problems that are harder than their corresponding decision versions.

### Results

In this paper we study the complexity of counting linear extensions when the parameter is the treewidth—a fundamental graph parameter which has already found a plethora applications in many areas of computer science [17, 18, 29]. In particular, we settle the fixed-parameter (in)tractability of the problem when parameterizing by the treewidth of two of the most prominent graphical representations of posets, the cover graph (also called the Hasse diagram) and the incomparability graph.

Our main result then provides the first evidence that the problem does not allow for an fpt algorithm parameterized by the treewidth of the cover graph unless ${\textsf {FPT}}={{{\textsf {W}}}}{ {[1]}}$. We remark that this complements the XP algorithm of [22] and resolves an open problem recently posed in the Dagstuhl seminar on Exact Algorithms [21]. The result is based on a so-called *fpt turing reduction* from Equitable Coloring parameterized by treewidth [14], and combines a counting argument with a fine-tuned construction to link the number of linear extensions with the existence of an equitable coloring. To the best of our knowledge, this is the first time this technique has been used to show fixed-parameter intractability of a counting problem.

We complement this negative result by obtaining an fpt algorithm for the problem when the parameter is the treewidth of the incomparability graph of the poset. To this end, we use the so-called *combined graph* (also called the cover-incomparability graph [5]) of the poset, which is obtained from the cover graph by adding the edges of the incomparability graph. We employ a special normalization procedure on a decomposition of the incomparability graph to show that the treewidth of the combined graph must be bounded by the treewidth of the incomparability graph. Once this is established, the result follows by giving a formulation of the problem in *Monadic Second Order Logic* and applying an extension of Courcelle’s Theorem for counting.

### Organization of the Paper

The paper is organized as follows. Section 2 introduces the required preliminaries and notation. Section 3 is then dedicated to proving the fixed-parameter intractability of the problem when parameterized by the treewidth of the cover graph, and the subsequent Sect. 4 presents our positive results for the problem. Concluding notes are then provided in Sect. 5.

## Preliminaries

For standard terminology in graph theory, such as the notions of a graph, digraph, path, etc. we refer readers to [11]. Given a graph *G*, we let *V*(*G*) denote its vertex set and *E*(*G*) its edge set. The (open) neighborhood of a vertex $x \in V(G)$ is the set $\{y\in V(G):\{x,y\}\in E(G)\}$ and is denoted by *N*(*x*). The closed neighborhood *N*[*v*] of *v* is defined as $N(v)\cup \{v\}$. A path between two disjoint vertex sets $A,B\subseteq V(G)$ is a path with one endpoint in *A*, one endpoint in *B*, and all internal vertices disjoint from $A\cup B$. A set $X\subseteq V(G)$ is a separator in *G* if $G-X$ contains at least two connected components.

We use [*i*] to denote the set $\{0,1,\dots ,i\}$. The following fact about prime numbers will also be useful later.

### Fact 1

([6]) For any $n\ge 4$, the product of primes strictly between *n* and $n^2$ is at least $n!2^n$.

### Treewidth

A *tree-decomposition* of a graph *G* is a pair $(T, \mathcal {X}=\{X_{t} \}_{t\in V (T )})$, where *T* is a rooted tree whose every vertex *t* is assigned a vertex subset $X_{t} \subseteq V(G)$, called a *bag*, such that the following properties hold:

1. $$\cup _{t\in V(T)}X_{t}=V(G)$$,
2. for every $u\in V(G)$, the set $T_{u}=\{t\in V(T): u\in X_{t}\}$ induces a connected subtree of *T* (*monotonicity*), and
3. for each $uv\in E(G)$ there exists $t\in V(T)$ such that $u,v\in X_{t}$.

To distinguish between the vertices of the tree *T* and the vertices of the graph *G*, we will refer to the vertices of *T* as *nodes*; for brevity, we will also interchange *T* and *V*(*T*) when the context is clear. The *width* of the tree-decomposition $\mathcal {T}$ is $\max _{t\in T}|X_{t}|-1$. The *treewidth* of *G*, $tw (G)$, is the minimum width over all tree-decompositions of *G*.

In some cases, we will make use of a well-established canonical form of tree-decompositions. A tree-decomposition $\mathcal {T}=(T,\mathcal {X})$ is *nice* if *T* contains a root *r* (introducing natural ancestor-descendant relations in *T*) and the following conditions are satisfied:

- $X_r=\emptyset$ and $X_\ell =\emptyset$ for every leaf $\ell$ of *T*. In other words, all the leaves as well as the root contain empty bags.
- Every non-leaf node of *T* is of one of the following three types:
  - *Introduce node* A note *t* with exactly one child $t'$ such that $X_t=X_{t'}\cup \{v\}$ for some vertex $v\not \in X_{t'}$; we say that *v* is *introduced* at *t*. If $u\in X_{t'}$ and *uv* is an edge in *G*, then we also say that *uv* is *introduced* at *t*.
  - *Forget node* A note *t* with exactly one child $t'$ such that $X_t=X_{t'}{\setminus } \{w\}$ for some vertex $w\in X_{t'}$; we say that *w* is *forgotten* at *t*.
  - *Join node* A node *t* with two children $t_1$, $t_2$ such that $X_{t}=X_{t_1}=X_{t_2}$.

We note that there exists a polynomial-time algorithm that converts an arbitrary tree-decomposition into a nice tree-decomposition of the same width [23]. A path-decomposition is a tree-decomposition where each node of *T* has degree at most 2, and nice path-decompositions are nice tree-decompositions which do not contain join nodes. Nice path-decompositions can also be computed from standard path-decompositions in polynomial time while preserving width [23]. Observe that any path-decomposition can be fully characterized by the order of appearance of its bags along *T*, and hence we will consider succinct representations of path-decompositions in the form $\mathcal {Q}=(Q_{1},\dots ,Q_{d})$, where $Q_i$ is the *i*-th bag in $\mathcal {Q}$. The *pathwidth* of *G*, $pw (G)$, is the minimum width of a path-decomposition of *G*.

We list some useful facts about treewidth and pathwidth.

#### Fact 2

[3, 4] There exists an algorithm which, given a graph *G* and an integer *k*, runs in time $\mathcal {O}(k^{\mathcal {O}(k^3)}n)$ and either outputs a tree-decomposition of *G* of width at most *k* or correctly identifies that $tw (G)>k$. Furthermore, there exists an algorithm which, given a graph *G* and an integer *k*, runs in time $\mathcal {O}(k^{\mathcal {O}(k^3)}n)$ and either outputs a path-decomposition of *G* of width at most *k* or correctly identifies that $pw (G)>k$.

#### Fact 3

(Folklore) Let $\mathcal {T}$ be a tree-decomposition of *G* and $t\in T$. Then each connected component of $G-X_t$ lies in a single subtree of $T-t$. In particular, for each connected component *C* of $G-X_t$ there exists a subtree $T'$ of $T-t$ such that for each vertex $a\in C$ there exists $t_a\in T'$ such that $a\in X_{t_a}$.

We note that if *G* is a directed graph, then $tw (G)$ and a tree-decomposition of *G* refer to the treewidth and a tree-decomposition of the underlying undirected graph of *G*, i.e., the undirected graph obtained by replacing each directed edge with an edge (and removing duplicate edges).

### Monadic Second Order Logic

We consider *Monadic Second Order* (MSO) logic on (edge-)labeled directed graphs in terms of their incidence structure whose universe contains vertices and edges; the incidence between vertices and edges is represented by a binary relation. We assume an infinite supply of *individual variables*$x,x_1,x_2,\dots$ and of *set variables*$X,X_1,X_2,\dots$ The *atomic formulas* are *Vx* (“*x* is a vertex”), *Ey* (“*y* is an edge”), *Ixy* (“vertex *x* is incident with edge *y*”), *Hxy* (“vertex *x* is the head of the edge *y*”), *Txy* (“vertex *x* is the tail of the edge *y*”), $x=y$ (equality), $x\ne y$ (inequality), $P_a x$ (“vertex or edge *x* has label *a*”), and *Xx* (“vertex or edge *x* is an element of set *X*”). *MSO formulas* are built up from atomic formulas using the usual Boolean connectives $(\lnot ,\wedge ,\vee ,\rightarrow ,\leftrightarrow )$, quantification over individual variables ($\forall x$, $\exists x$), and quantification over set variables ($\forall X$, $\exists X$).

Let $\varPhi (X)$ be an MSO formula with a free set variable *X*. For a labeled graph $G=(V,E)$ and a set $S\subseteq E$ we write $G \models \varPhi (S)$ if the formula $\varPhi$ holds true on *G* whenever *X* is instantiated with *S*.

The following result (an extension of the well-known Courcelle’s Theorem [8]) shows that if *G* has bounded treewidth then we can count the number of sets *S* with $G \models \varPhi (S)$.

#### Fact 4

[1] Let $\varPhi (X)$ be an MSO formula with a free set variable *X* and *w* a constant. Then there is a linear-time algorithm that, given a labeled directed graph $G=(V,E)$ of treewidth at most *w*, outputs the number of sets $S \subseteq E$ such that $G \models \varPhi (S)$.

We note that the above result requires a tree-decomposition of width at most *w* to be provided with the input. However, as seen in Fact 2, for a graph of treewidth at most *w* such a tree decomposition can be found in linear time, hence we can drop this requirement from the statement of the theorem.

### Posets

A *partially ordered set* (*poset*) $\mathcal {P}$ is a pair $(P,\le ^P)$ where *P* is a set and $\le ^P$ is a reflexive, antisymmetric, and transitive binary relation over *P*. The *size* of a poset $\mathcal {P}=(P,\le ^P)$ is $|{\mathcal P}|:=|P|$. We say that *pcovers*$p'$ for $p,p' \in P$, denoted by $p' \lhd ^P p$, if $p' \le ^P\! p$, $p \ne p'$, and for every $p''$ with $p' \le ^P\! p''\le ^P\! p$ it holds that $p''\in \{p,p'\}$. We say that *p* and $p'$ are *incomparable* (in $\mathcal {P}$), denoted $p \parallel ^P p'$, if neither $p \le ^P\! p'$ nor $p' \le ^P\! p$.

A *chainC* of $\mathcal {P}$ is a subset of *P* such that $x \le ^P y$ or $y \le ^P x$ for every $x,y \in C$. An *antichainA* of $\mathcal {P}$ is a subset of *P* such that for all $x,y \in A$ it is true that $x \parallel ^P y$. A family $C_1,\dots ,C_\ell$ of pairwise disjoint subsets of *P* forms a *total order* if for each $i,j\in [\ell ]$ and each $a\in C_i$, $b\in C_j$, it holds that $a\le b$ iff $i<j$. Furthermore, for each $i\in [\ell -1]$ we say that $C_i$ and $C_{i+1}$ are *consecutive*. We call a poset $\mathcal {P}$ such that every two elements of $\mathcal {P}$ are comparable a *linear order*. A *linear extension* of a poset $\mathcal {P}= (P,\le ^P)$ is a reflexive, antisymmetric, and transitive binary relation $\preceq$ over *P* such that $x\preceq y$ whenever $x\le ^P y$ and the poset $\mathcal {P}^*=(P, \preceq )$ is a linear order.

We denote the number of linear extensions of $\mathcal {P}$ by $e(\mathcal {P})$. For completeness, we provide a formal definition of the problem of counting the number of linear extensions below. 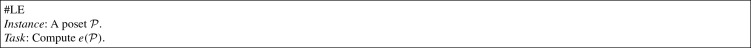


We consider the following graph representations of a poset $\mathcal {P}=(P,\le ^\mathcal {P})$. The *cover graph* of $\mathcal {P}$, denoted $C(\mathcal {P})$, is the undirected graph with vertex set *P* and edge set $\{\{a,b\}~|~a\lhd b\}$. The *incomparability graph* of $\mathcal {P}$, denoted $I(\mathcal {P})$, is the undirected graph with vertex set *P* and edge set $\{\{a,b\}~|~a\parallel b\}$. The *combined graph* of $\mathcal {P}$, denoted $I_C(\mathcal {P})$, is the directed graph with vertex set *P* and edge set $\{(a,b)~|~(a\lhd b)\vee (a\parallel b)\}$. Finally, the *poset graph* of $\mathcal {P}$, denoted $P_G(\mathcal {P})$, is the directed graph with vertex set *P* and edge set $\{(a,b)~|~a\le b\}$. We will use the following known fact about tree-decompositions and path-decompositions of incomparability graphs.

#### Fact 5

[20, Theorem 2.1] Let $\mathcal {P}$ be a poset. Then $tw (I(\mathcal {P})) = pw (I(\mathcal {P}))$.

#### Corollary 1

(of Facts 2 and 5) Let $\mathcal {P}$ be a poset and $k=tw (I(\mathcal {P}))$. Then it is possible to compute a nice path-decomposition $\mathcal {Q}$ of $I(\mathcal {P})$ of width at most *k* in time $\mathcal {O}(k^{\mathcal {O}(k^3)}n)$.

### Parameterized Complexity

We refer the reader to [9, 12, 16] for an in-depth introduction to parameterized complexity; here we only briefly summarize the most important notions required by our results.

A *parameterized counting problem*$\mathcal {A}$ is a function $\varSigma ^* \times \mathbb {N}\rightarrow \mathbb {N}$ for some finite alphabet $\varSigma$. We call a parameterized counting problem $\mathcal {A}$*fixed-parameter tractable* (${\textsf {FPT}}$) if $\mathcal {A}$ can be computed in time $f(k) \cdot |x|^{\mathcal {O}(1)}$ where *f* is an arbitrary computable function and (*x*, *k*) is the instance. The complexity class W[1] can be seen an the analog of NP for parameterized decision problems. To prove that a parameterized problem $\mathcal A$ is W[1]-hard, one can give an *fpt turing reduction* from a known W[1]-hard problem $\mathcal B$ to $\mathcal A$; such an fpt turing reduction is a deterministic algorithm solving $\mathcal A$ with an oracle to $\mathcal B$ with the following properties: (a) the algorithm is FPT, and (b) the parameter for $\mathcal B$ in each oracle query is bounded by a function of the parameter for $\mathcal A$. To avoid confusion, we remark that there also exists the complexity class $\#$W[1] which is an analog of $\#$P for parameterized counting problems.

Our main negative result is based on an fpt turing reduction from the following fairly well-known W[1]-hard decision problem [14]. 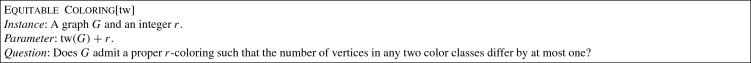


We denote by $\#EC (G,r)$ the number of equitable colorings of graph *G* with *r* colors. We remark that the problem remains W[1]-hard even if we restrict to the instances, where |*V*(*G*)| is divisible by *r*. This can be seen, for example, by padding of the instance by a single isolated clique.

## Fixed-Parameter Intractability of Counting Linear Extensions

The goal of this section is to prove Theorem 1, stated below.

### Theorem 1

#LE parameterized by the treewidth of the cover graph of the input poset does not admit an fpt algorithm unless W[1]=${\textsf {FPT}}$.

We begin by giving a brief overview of the proof, whose general outline follows the #P-hardness proof of the problem [6]. However, since our parameter is treewidth, we needed to reduce from a problem that is not fixed-parameter tractable parameterized by treewidth. Consequently, instead of reducing from SAT, we will use Equitable Coloring. This made the reduction considerably more complicated and required the introduction of novel gadgets, which allow us to encode the problem without increasing the treewidth too much.

The proof is based on solving an instance (*G*, *r*) of Equitable Coloring[tw] in FPT time using an oracle that solves #LE in FPT time parameterized by the treewidth of the cover graph (i.e., an fpt turing reduction). The first step is the construction of an auxiliary poset $\mathcal {P}(G,r)$ of size $2(r-1)|V(G)|+(r^2-1)|E(G)|$. Then, for a given sufficiently large (polynomially larger than |*V*(*G*)|) prime number *p*, we show how to construct a poset $\mathcal {P}(G,r,p)$ such that $e(\mathcal {P}(G,r,p))\equiv e(\mathcal {P}(G,r))\cdot \#EC (G,r)\cdot A_p \mod p$, where $A_p$ is a constant that depends on *p* and is not divisible by *p*. Therefore, if we choose a prime *p* that does not divide $e(\mathcal {P}(G,r))\cdot \#EC (G,r)$, then $e(\mathcal {P}(G,r,p))$ will not be divisible by *p*. Using Fact 1 we show that if $\#EC (G,r)\ne 0$, then there always exists a prime *p* within a specified polynomial range of |*V*| such that *p* does not divide $e(\mathcal {P}(G,r))\cdot \#EC (G,r)$.

From the above, it follows that there exists an equitable coloring of *G* with *r* colors if and only if, for at least one prime *p* within a specified (polynomial) number range, the number of linear extensions of $\mathcal {P}(G,r,p)$ is not divisible by *p*. Moreover, we show that all inputs for the oracle will have size polynomial in the size of *G* and treewidth bounded by a polynomial in $tw (G)+r$. Before proceeding to a formal proof of Theorem 1, we state two auxiliary lemmas which will be useful for counting linear extensions later in the proof.

### Lemma 1

If a poset $\mathcal {P}$ is a disjoint union of posets $\mathcal {P}_1,\dots ,\mathcal {P}_k$ for some positive integer *k*, then

$$\begin{aligned} e(\mathcal {P})={{\left( \sum _{i=1}^k |\mathcal {P}_i|\right) !} \over {\prod _{i=1}^k |\mathcal {P}_i|!}}\prod _{i=1}^k e(\mathcal {P}_i) . \end{aligned}$$

### Proof

We use induction on *k*, and observe that the lemma trivially holds for $k=1$. Let $\mathcal {Q}$ denote the disjoint union of posets $\mathcal {P}_1,\dots ,\mathcal {P}_{k-1}$. For each combination of linear extension of $\mathcal {Q}$ and of $\mathcal {P}_k$ there are ${{|\mathcal {Q}|+|\mathcal {P}_k|}\atopwithdelims (){|\mathcal {P}_k|}}$ linear extensions of $\mathcal {P}$. Hence,

$$\begin{aligned} e(\mathcal {P})= & {} e(\mathcal {Q})e(\mathcal {P}_k){{|\mathcal {Q}|+|\mathcal {P}_k|}\atopwithdelims (){|\mathcal {P}_k|}} = {{(\sum _{i=1}^{k-1} |\mathcal {P}_i|)!} \over {\prod _{i=1}^{k-1} |\mathcal {P}_i|!}}\bigg (\prod _{i=1}^{k-1} e(\mathcal {P}_i)\bigg )\cdot e(\mathcal {P}_k) \cdot {{\sum _{i=1}^k |\mathcal {P}_i|}\atopwithdelims (){|\mathcal {P}_k|}}\\= & {} {{(\sum _{i=1}^{k-1} |\mathcal {P}_i|)!} \over {\prod _{i=1}^{k-1} |\mathcal {P}_i|!}}{{(\sum _{i=1}^k |\mathcal {P}_i|)!}\over {(\sum _{i=1}^{k-1} |\mathcal {P}_i|)!|\mathcal {P}_k|!}}\prod _{i=1}^{k} e(\mathcal {P}_i)= {{(\sum _{i=1}^k |\mathcal {P}_i|)!} \over {\prod _{i=1}^k |\mathcal {P}_i|!}}\prod _{i=1}^k e(\mathcal {P}_i). \end{aligned}$$

$$\square $$

In the following we say that a set *C* of elements of a poset $\mathcal {P}$ is a connected component of $\mathcal {P}$ if *C* is a connected component of $C(\mathcal {P})$.

### Lemma 2

Let *p* be a prime number and $\mathcal {Q}$ be a connected component of a poset $\mathcal {P}$ such that $|\mathcal {Q}|=p-1$. If the number of linear extensions of $\mathcal {P}$ is not divisible by *p*, then the number of elements in each connected component of $\mathcal {P}$ other than $\mathcal {Q}$ is divisible by *p*.

### Proof

Let $\mathcal {P}_1$ be a connected component of $\mathcal {P}$ different than $\mathcal {Q}$. First note that from Lemma 1, it is clear that $e(\mathcal {P})$ will be divisible by the number of linear extensions of the poset $\mathcal {P}'$ formed as a disjoint union of $\mathcal {Q}$ and $\mathcal {P}_1$. Now, by Lemma 1 it holds that

$$\begin{aligned} e(\mathcal {P}')={{(p-1+|\mathcal {P}_1|)!} \over {(p-1)!|\mathcal {P}_1|!}} e(\mathcal {P}_1)e(\mathcal {Q}). \end{aligned}$$

Since $e(\mathcal {P})$ is not divisible by *p*, it must follow that $e(\mathcal {P}')$ is also not divisible by *p*. Note that $${{(p-1+|\mathcal {P}_1|)!} \over {(p-1)!|\mathcal {P}_1|!}}=\left( {\begin{array}{c}p-1+|\mathcal {P}_1|\\ p-1\end{array}}\right) $$ is a natural number and a divisor of $e(\mathcal {P}')$. Furthermore, $(p-1)!$ cannot be divisible by *p* since *p* is prime. Hence it follows that $${{(p-1+|\mathcal {P}_1|)!} \over {|\mathcal {P}_1|!}} = \left( {\begin{array}{c}p-1+|\mathcal {P}_1|\\ p-1\end{array}}\right) \cdot (p-1)!$$ cannot be divisible by *p*. Suppose that $|\mathcal {P}_1|=ap+b$ for some non-negative integers *a* and *b* such that $b<p$; then we obtain that the expression ${{(p-1+ap+b)!} \over {(ap+b)!}}$ is not divisible by *p*. But

$$\begin{aligned} {{(p-1+ap+b)!} \over {(ap+b)!}} = \prod _{i=1}^{p-1}\left( ap+b+i\right) , \end{aligned}$$

which is clearly divisible by $(a+1)p$ whenever $b\ge 1$. Therefore $b=0$ and hence $|\mathcal {P}_1|$ is divisible by *p*, which concludes the proof of the lemma. $\square$

We now proceed to the proof of our main theorem.

### Proof

(of Theorem 1) The proof is structured as follows. We begin by giving the construction of $\mathcal {P}(G,r)$ and $\mathcal {P}(G,r,p)$, after which we establish the desired properties of $\mathcal {P}(G,r,p)$ and $\mathcal {P}(G,r)$, and summarize in the conclusion.

**Fig. 1:**
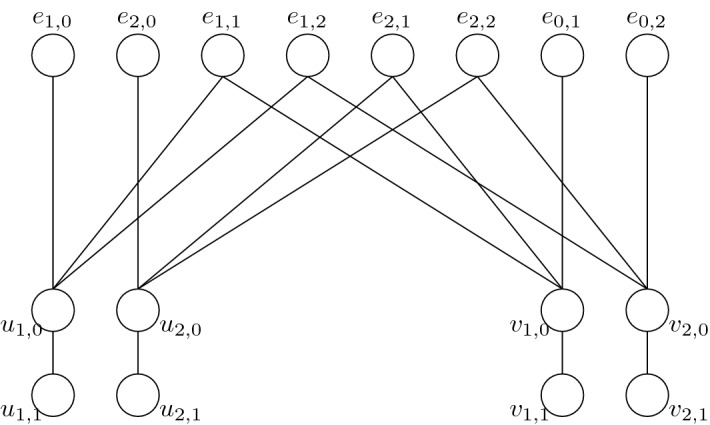
The cover graph for an edge $e=uv$ of *G* in $\mathcal {P}(G,3)$

*Construction of*$\mathcal {P}(G,r)$*and the main gadget* Let (*G*, *r*) be an instance of Equitable Coloring[tw] such that |*V*(*G*)| is divisible by *r* (if this is not the case, then this can be enforced by padding the instance with isolated vertices, see also [14]). We begin by constructing the poset $\mathcal {P}(G,r)$, which will play an important role later on. For every vertex *v* of *V*(*G*) we create $2(r-1)$ elements denoted $v_{i,j}$, where $1\le i\le r-1$ and $j \in \{0,1\}$, such that the only dependencies in the poset between these elements are $v_{i,1} \le v_{i,0}$ for all $v\in V(G)$, for all $i\in \{1,\dots , r-1\}$. For every edge $e=uv\in E(G)$ we create $r^2-1$ pairwise-incomparable elements $e_{i,j}$, such that $(i,j)\in (\{0,\dots ,r-1\}^2{\setminus } \{(0,0)\})$. The dependencies of $e_{i,j}$ are: if $i>0$ then $u_{i,0} \le e_{i,j}$, and if $j>0$ then $v_{j,0} \le e_{i,j}$ (see also Fig. 1).

**Fig. 2:**
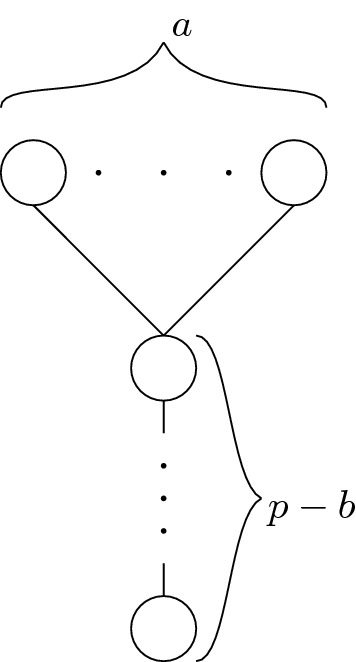
An (*a*, *b*)-flower

*Construction of*$\mathcal {P}(G,r,p)$ Let us now fix a prime number *p* such that *p* does not divide $e(\mathcal {P}(G,r))$ and $p>2r|V(G)|+r^2|E(G)|$. The main gadget in our reduction is a so-called (*a*, *b*)-*flower*, which consists of an antichain of *a* vertices (called the *petals*) covering a chain of $p-b$ elements (called the *stalk*); an illustration is provided in Fig. 2. Due to Lemma 2, (*a*, *b*)-flowers will later allow us to force a choice of exactly *b* vertices out of *a*.

Let *G* be a graph, *r* be an integer and *p* be a prime number as above. Recall that |*V*(*G*)| is divisible by *r* and let $s={{|V(G)|}\over {r}}$ (note that this implies that each color in an equitable coloring of *G* must occur precisely *s* times in *G*). We proceed with a description of the poset $\mathcal {P}(G,r,p)$. The poset $\mathcal {P}(G,r,p)$ is split into $r+3$ “*levels*” $L_1,\dots ,L_{r+3}$ by linearly ordered elements $a_0\le a_1\le \dots \le a_{r+2} \le a_{r+3}$, called the *anchors*. Each of these levels, besides $L_{r+3}$, will consist of some flowers and a chain of $p-1$ elements which we call a *stick*; each of these flowers and the stick will always be pairwise incomparable. The anchors $a_0$ and $a_{r+3}$ are the unique minimum and maximum elements, respectively. The stick and all the stalks of flowers in level $L_{i}$ will always lie between two consecutive elements $a_{i-1}$ and $a_{i}$, and the petals of these flowers will be incomparable with $a_i$ as well as some anchors above that (as defined later). Observe that while the relative position of any stalk and any anchor is fixed in every linear extension, petals can be placed above $a_i$.

**Fig. 3:**
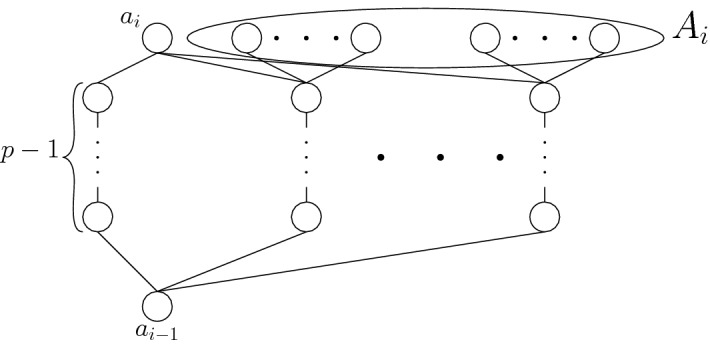
Each level consists of a chain of length $p-1$ and a few flowers. The set of petals associated with level $L_{i}$ is denoted by $A_{i}$

We say that a flower (or its stalk, petals, or elements) is *associated* with the level in which it is constructed, i.e., with the level $L_i$ such that $a_{i-1}\le c\le a_i$ for stalk elements *c* and $a_{i-1}\le d$ and $d\parallel a_i$ for petals *d*. We denote the set of all petals associated with level $L_i$ as $A_i$ (see Fig. 3). For the construction, it will be useful to keep in mind the following intended goal: whenever an (*a*, *b*)-flower is placed in level *i*, it will force the selection of precisely *b* petals (from its total of *a* petals), where selected elements remain on level *i* (i.e., between $a_{i-1}$ and $a_i$) in the linear extension and unselected elements are moved to level $r+2$ (i.e., between $a_{r+2}$ and $a_{r+3}$) in the linear extension. We will later show that the total number of linear extensions which violate this goal must be divisible by *p*, and hence such extensions can all be disregarded modulo *p*.

The first *r* levels are so-called *color class* levels, each representing one color class. We use these levels to make sure that every color class contains exactly *s* vertices. Aside from the stick, each such level contains a single (|*V*(*G*)|, *s*)-flower. Recall that the stalk and the stick on level $1\le i\le r$ both lie between anchors $a_{i-1}$ and $a_i$, and that the stalk and the flower are incomparable. We associate each petal of the flower at level $L_i$ with a unique vertex $v\in V(G)$ and denote the petal $v_i$. Each petal $v_i$ will be incomparable with all anchors above $a_{i-1}$ up to $a_{r+3}$, i.e., $v_i\parallel a_j$ for $i\le j\le r+2$ and $v_i\le a_{r+3}$. Intuitively, the flower in each color class level will later force a choice of *s* vertices to be assigned the given color.

Level $L_{r+1}$ is called the *vertex* level and consists of one stick and |*V*(*G*)|-many (*r*, 1)-flowers; the purpose of this level is to ensure that every vertex is assigned exactly one color. Each flower is associated with one vertex $v\in V(G)$ and we denote the petals of the flower associated with vertex *v* as $v^i$ for $1\le i\le r$. We set $v_i \le v^i$ for all $v\in V(G)$ and $1\le i\le r$.

Level $L_{r+2}$ is called the *edge* level, and its purpose is to ensure that the endpoints of every edge have a different color. It consists of a stick and |*E*(*G*)|-many $(r^2,1)$-flowers. Each flower is associated with one edge $e=uv\in V(G)$ and we denote the petals of the flower associated with *e* as $e_{i,j}$ for $1\le i\le r$ and $1\le j\le r$. Moreover, for edge $e=uv$ we set $u^i \le e_{i,j}$, $v^j\le e_{i,j}$, and we set $a_{r+2} \le e_{i,j}$ whenever $i=j$. Observe that this forces any petal $e_{i,i}$ to lie between $a_{r+2}$ and $a_{r+3}$ in every linear extension (i.e., prevents $e_{i,i}$ from being “selected”).

Level $L_{r+3}$ is called the *trash level*. It does not contain any new elements in the poset, but it plays an important role in the reduction: we will later show that any petals which are interpreted as “not selected” must be located between $a_{r+2}$ and $a_{r+3}$ in any linear extension that is not automatically “canceled out” due to counting modulo *p*.

**Fig. 4:**
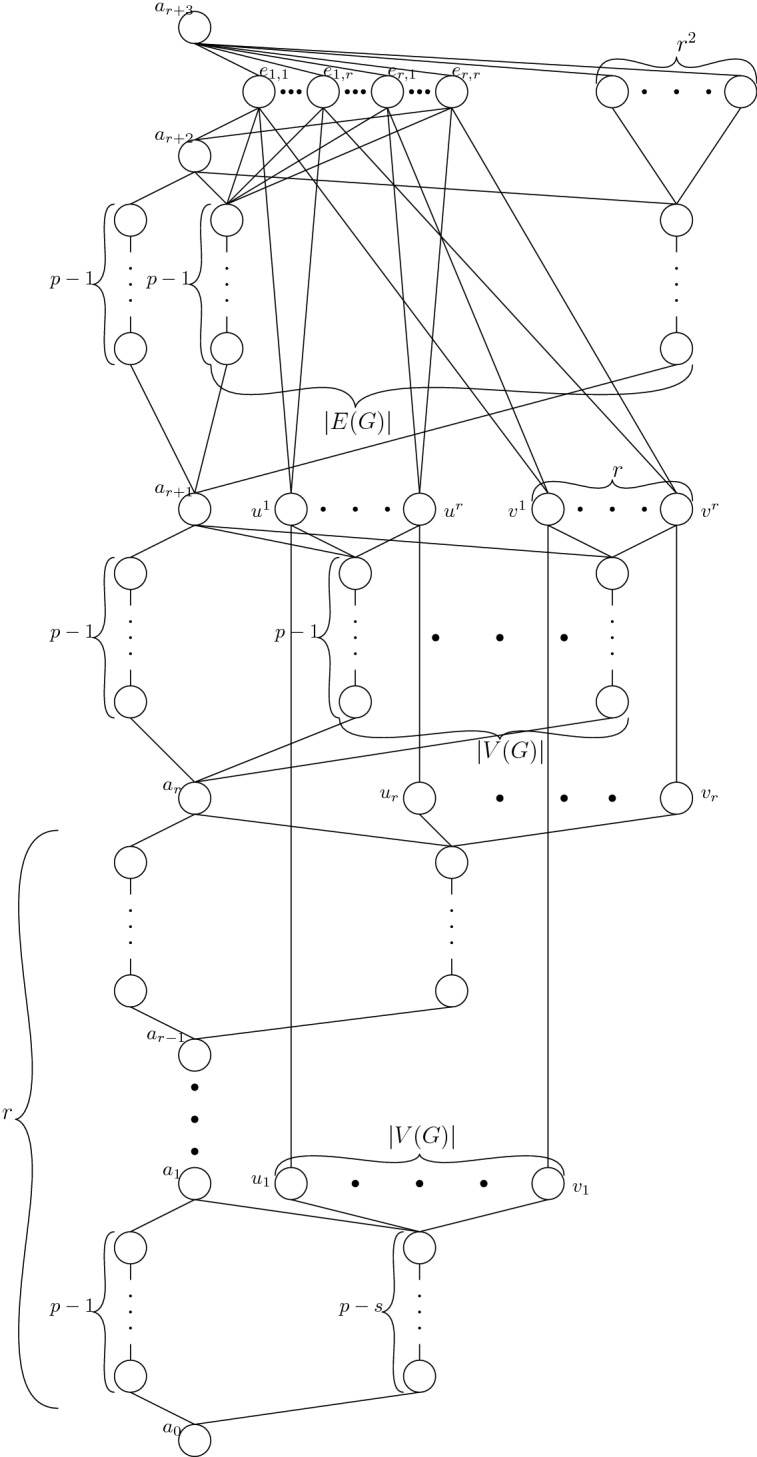
The cover graph of $\mathcal {P}(G,r,p)$. The edge *e* is the edge in *G* between vertices *u* and *v*

A high-level overview of the whole constructed poset $\mathcal {P}(G,r,p)$ is presented in Fig. 4.

*Establishing the desired properties of*$\mathcal {P}(G,r,p)$*and*$\mathcal {P}(G,r)$ We begin by formalizing the notion of selection. Let a *configuration* be a partition $\phi$ of petals of all flowers into $r+3$ sets $L_1^\phi , \dots , L_{r+3}^\phi$. Let $\varPhi$ denote a set of all configurations. We say that a linear extension $\preceq$ of $\mathcal {P}(G,r,p)$*respects* the configuration $\phi$ if $L_1^\phi \preceq a_1\preceq L_2^\phi \preceq a_2\preceq \dots \preceq a_{r+2}\preceq L_{r+3}^\phi$ and we denote the set of all linear extensions of $\mathcal {P}(G,r,p)$ that respects $\phi$ by $\mathcal {L}^\phi$. We say that a configuration $\phi$ is *consistent* if $\mathcal {L}^\phi$ is non-empty; this merely means that $L_1^\phi \le a_1\le L_2^\phi \le a_2\le \dots \le a_{r+2}\le L_{r+3}^\phi$ does not violate any inequalities in $\mathcal {P}(G,r,p)$. Observe that if $\phi$ is consistent, then $\mathcal {L}^\phi$ is exactly the set of linear extension of the partial order $\mathcal {P}^\phi (G,r,p)$, where $\mathcal {P}^\phi (G,r,p)$ is obtained by enriching $\mathcal {P}(G,r,p)$ with the relations $L_1^\phi \le a_1\le L_2^\phi \le a_2\le \dots \le a_{r+2}\le L_{r+3}^\phi$ and performing transitive closure (in other words, $\mathcal {P}^\phi (G,r,p)$ is obtained by enforcing $\phi$ onto $\mathcal {P}(G,r,p)$).

Since every linear extension of $\mathcal {P}(G,r,p)$ respects exactly one configuration, it is easy to see that $e(\mathcal {P}(G,r,p)) = \sum _{\phi \in \varPhi } |\mathcal {L}^\phi | = \sum _{\phi \in \varPhi } e(\mathcal {P}^\phi (G,r,p))$. Intuitively, a configuration $\phi$*contributes* to the above sum modulo *p* if $e(\mathcal {P}^\phi (G,r,p))$ is not divisible by *p*. We shall prove that the only configurations which contribute to this sum modulo *p* are those where from every (*a*, *b*)-flower there are exactly *b* petals in the same level as the stalk, and the remaining $a-b$ petals are in the trash. Furthermore, in each configuration $\phi$ which contributes to the above sum modulo *p*, the petals in $L_{r+1}^\phi$ represent a proper equitable coloring of *G* with *r* colors, and each such configuration is respected by the same number of linear extensions.

Let us first remark that for any configuration $\phi$, the anchors $a_0,a_1,\dots ,a_{r+3}$ are comparable to all elements of $\mathcal {P}^\phi (G,r,p)$. Now, let $\mathcal {P}_{L_i}^\phi$ be the poset induced by all elements $e\in \mathcal {P}^\phi (G,r,p)$ such that $a_{i-1}\le e\le a_i$ and $e\ne a_{i-1}$, $e\ne a_i$. It is readily seen that $e(\mathcal {P}^\phi (G,r,p)) = \prod _{i=1}^{r+3}e(\mathcal {P}_{L_i}^\phi )$. We proceed by stating a series of claims about our construction.

### Claim 1

For each $i\in \{1,\dots , r\}$, it holds that either $e(\mathcal {P}_{L_i}^\phi ) \equiv 0 \mod p$, or $e(\mathcal {P}_{L_i}^\phi ) = s!{{2p-1} \atopwithdelims (){p}}$ and $L_i^\phi$ contains exactly *s* petals of $A_i$ and no other petals.

### Proof

(of the Claim) Assume that $e(\mathcal {P}_{L_i}^\phi ) \not \equiv 0 \mod p$ and recall that level $L_i$ contains a stick, which is a chain of $p-1$ elements that is incomparable with all elements of $\mathcal {P}_{L_i}^\phi$ in every configuration $\phi$. By Lemma 2 this implies that every connected component of $\mathcal {P}_{L_i}^\phi$ distinct from the stick has size divisible by *p*. Clearly, $L_i^\phi$ contains only those stalks that are associated with the level $L_i$, and it contains all such stalks. It is readily seen from the construction that any petal in $\cup _{j<i}A_j$ would necessarily form a component of size one in $\mathcal {P}_{L_i}^\phi$. Hence, $\mathcal {P}_{L_i}^\phi$ contains only elements associated with level $L_i$, namely elements of the stick and elements of a (|*V*(*G*)|, *s*)-flower. Moreover, by Lemma 2 and the fact that $|V(G)|+p-s<2p$, each such flower has exactly *p* elements in level $\mathcal {P}_{L_i}^\phi$. Since the $p-s$ elements of the stalk must be in $\mathcal {P}_{L_i}^\phi$, the poset $\mathcal {P}_{L_i}^\phi$ contains exactly *s* elements of $A_i$. Clearly, the number of linear extensions of the petals of the (|*V*(*G*)|, *s*)-flower in $\mathcal {P}_{L_i}^\phi$ is *s*! and hence by Lemma 1$e(\mathcal {P}_{L_i}^\phi ) = s!{{2p-1} \atopwithdelims (){p}}$, which concludes the proof. $\square$

### Claim 2

Either $e(\mathcal {P}_{L_{r+1}}^\phi ) \equiv 0 \mod p$, or $e(\mathcal {P}_{L_{r+1}}^\phi ) = {{(|V(G)|p+p-1)!} \over {(p-1)}!(p!)^{|V(G)|}}$ and $L_{r+1}^\phi$ contains exactly |*V*(*G*)| elements of $A_{r+1}$, specifically one petal for each (*r*, 1)-flower on level $L_{r+1}$.

### Proof

(of the Claim) Assume $e(\mathcal {P}_{L_{r+1}}^\phi ) \not \equiv 0 \mod p$, and let us first examine elements that are not associated with level $L_{r+1}$. Clearly, no element associated with level $L_{r+2}$ can appear in $\mathcal {P}_{L_{r+1}}^\phi$ and the only elements associated with any level $i<r+1$ that can end up in $\mathcal {P}_{L_{r+1}}^\phi$ are petals. Each of these elements is smaller than exactly one petal at level $L_{r+1}$ and incomparable to all other elements associated with this level. It is easy to see that largest possible size of a connected component of $\mathcal {P}_{L_{r+1}}^\phi$ is $p-1+2r<2p$. By Lemma 2, every connected component in $\mathcal {P}_{L_{r+1}}^\phi$ (except for the stick) will have size *p*, and therefore $\mathcal {P}_{L_{r+1}}^\phi$ will contain exactly one element for every set of petals associated with $L_{r+1}$ and no other elements. Hence, $\mathcal {P}_{L_{r+1}}^\phi$ consists of |*V*(*G*)| chains of length *p* and one chain of length $p-1$. Then $e(\mathcal {P}_{L_{r+1}}^\phi ) = {{(|V(G)|p+p-1)!} \over {(p-1)}!(p!)^{|V(G)|}}$ follows from Lemma 1. $\square$

### Claim 3

Either $e(\mathcal {P}_{L_{r+2}}^\phi ) \equiv 0 \mod p$, or $e(\mathcal {P}_{L_{r+2}}^\phi ) = {{(|E(G)|p+p-1)!} \over {(p-1)}!(p!)^{|E(G)|}}$ and $L_{r+2}^\phi$ contains exactly |*E*(*G*)| elements of $A_{r+2}$, specifically one petal for each $(r^2,1)$-flower on level $L_{r+2}$.

### Proof

(of the Claim) The idea of the proof is similar to the proof of the previous claim, with one additional obstacle: that several flowers can be connected with petals from lower levels into one connected component on level $L_{r+2}$ through the petals of flowers on level $L_{r+1}$. So, assume $e(\mathcal {P}_{L_{r+2}}^\phi )$ contains a connected component *C* which contains at least a single stalk. If *C* contains precisely the single stalk, then by Lemma 2 we have $e(\mathcal {P}_{L_{r+2}}^\phi ) \equiv 0 \mod p$. Otherwise, for each stalk in *C*, there must be at least one petal in the same flower (otherwise the stalk cannot be connected to the rest of *C*); in other words, the intersection of each flower and *C* contains at least *p* vertices. Let *a* denote the number of flowers which intersect *C*, $b_2$ denote $|A_{r+2}\cap C|$, $b_1$ denote $|A_{r+1}\cap C|$ and $b_0$ denote $\sum _{i=1}^r|A_r\cap C|$. Then it follows that $|C|=p\cdot a+(b_2-a)+b_1+b_0\le p\cdot a+r^2|E|+r|V|+r|V|$, and recall that $r^2|E|+r|V|+r|V|<p$. Furthermore, if $b_1>0$ (and at least one petal from $A_{r+1}$ is required unless *C* contains only a single flower), we have $a\cdot p<|C|<(a+1)\cdot p$. Hence any such *C* cannot have size divisible by *p* and by Lemma 2 we have $e(\mathcal {P}_{L_{r+2}}^\phi ) \equiv 0 \mod p$. Otherwise, if no two flowers are connected through a petal of a flower associated with level $L_{r+1}$, then every connected component of $\mathcal {P}_{L_{r+2}}^\phi$ of size *p* must consist of a stalk and exactly one petal and the claim follows analogously as the proof of Claim 2. $\square$

### Claim 4

If $\phi$ is a consistent configuration and for all $i\in \{1,\dots , r+2\}$ it holds that $e(\mathcal {P}_{L_{i}}^\phi ) \not \equiv 0 \mod p$, then *1)* the petals in $L_{r+1}^\phi$ encode a proper equitable coloring of *V*(*G*) where vertex *v* receives color *i* iff the petal $v_i$ lies in $L_i^\phi$, and *2)*$\mathcal {P}_{L_{r+3}}^\phi$ is isomorphic with $\mathcal {P}(G,r)$.

### Proof

(of the Claim) From Claims 1, 2 and 3 together with the assumption that $e(\mathcal {P}_{L_{i}}^\phi ) \not \equiv 0 \mod p$, it follows that each of the levels $L_1^\phi ,\dots ,L_{r}^\phi$ contains exactly *s* petals associated with the corresponding level, level $L_{r+1}^\phi$ contains exactly one petal for each vertex of *G* and level $L_{r+2}^\phi$ contains exactly one petal for each edge of *G*.

For the first part of this claim, we observe that each pair of petals in $L_1^\phi ,\dots , L_r^\phi$ are associated with distinct vertices of *G*. If this were not the case, then since $|V(G)|=rs$ there would exist a vertex *v* such that no element of $L_1^\phi ,\dots , L_r^\phi$ is associated with *v*. But due to the construction at level $r+1$ there exists some $i\in \{1,\dots ,r\}$ such that $v^i\in L_{r+1}^\phi$. Then, since $v_i\le v^i$ and $v_i$ can only occur either in level $L_{i}^\phi$ or $L_{r+3}^\phi$ (the latter of which lies above $v^i$ in the linear extension due to the configuration $\phi$), this would lead to a contradiction. In particular, we conclude that there is a matching between the petals in level $r+1$ (encoding the color for each vertex) and the union of petals in levels $1,2,\dots r$ (encoding the vertices assigned to each color class), and by Claim 1 it follows that there are exactly *s* petals in $L_{r+1}$ associated with each color class.

We now argue that the coloring is proper. Observe that by the same argument as above, if an edge $e=uv$ satisfies $e_{i,j}\in L_{r+2}^\phi$, then $u^i\in L_{r+1}^\phi$ and $v^j\in L_{r+1}^\phi$. From the construction of $\mathcal {P}(G,r,p)$ it follows that if $i=j$, then $e_{i,j}\not \in L_{r+2}$. Combining these two facts we get that the coloring encoded in $L_{r+1}^\phi$ is indeed proper.

Now let us take a look at level $L_{r+3}^\phi$. To prove the claim, we will construct an isomorphism *f* from elements of $\mathcal {P}_{L_{r+3}}^\phi$ to elements of $\mathcal {P}(G,r)$. For every vertex $v\in V(G)$, precisely one element $v^i\in L_{r+1}^\phi$ and precisely one of the first *r* levels contains an element associated with *v*; to be precise, $v_i\in L_{i}^\phi$ and $v_j\in L_{r+3}^\phi$ and hence also $v^j\in L_{r+3}^\phi$ for all $j\not = i$. We set $f(v^j)=v_{j,0}$ and $f(v_j)=v_{j,1}$, whenever $j\not = i$ and $j<r$. For the last remaining elements, we set $f(v^r)=v_{i,0}$ and $f(v_r)=v_{i,1}$. Next, for every edge $e=uv$ there is exactly one $e_{a,b}\in L_{r+2}^\phi$. Moreover, if $e_{a,b}\in L_{r+2}^\phi$ then $u^a\in L_{r+1}^\phi$ and $v^b\in L_{r+1}^\phi$, and all other petals for this edge *e* are in $L_{r+3}^\phi$. Let $g_i(r)=i$, $g_i(i)=0$, and $g_i(k)=k$ otherwise. Then we set $f(e_{i,j})=e_{g_a(i),g_b(j)}$. Observe that, since $e_{a,b}$ does not lie in $L_{r+3}^\phi$, no edge is mapped to the non-existent element $e_{0,0}$ in $\mathcal {P}(G,r)$. It is straightforward to verify that *f* is really bijective mapping between elements of $\mathcal {P}_{L_{r+3}}^\phi$ and $\mathcal {P}(G,r)$. Moreover, $f(u)\le f(v)$ in $\mathcal {P}(G,r)$ if and only if $u\le v$ in $\mathcal {P}_{L_{r+3}}^\phi$. Therefore, $\mathcal {P}_{L_{r+3}}^\phi$ is isomorphic with $\mathcal {P}(G,r)$ and the claim holds. $\square$

### Claim 5

$e(\mathcal {P}(G,r,p))\not \equiv 0 \mod p$ if and only if $e(\mathcal {P}(G,r))\cdot \#EC (G,r)\not \equiv 0 \mod p$.

### Proof

(of the Claim) From previous claims and in particular Claim 4, we already know that $e(\mathcal {P}^\phi (G,r,p))\ne 0 \mod p$ only if $\phi$ corresponds to an equitable coloring of *G* with *r* colors. Moreover, we know that $e(\mathcal {P}^\phi (G,r,p)) = \prod _{i=1}^{r+3}e(\mathcal {P}_{L_i}^\phi )$ and if $e(\mathcal {P}^\phi (G,r,p))\not \equiv 0 \mod p$, then

$$\begin{aligned} \prod _{i=1}^{r+3}e(\mathcal {P}_{L_i}^\phi ) = \left( s!{{2p-1} \atopwithdelims (){p}}\right) ^r {{(|V(G)|p+p-1)!} \over {(p-1)}!(p!)^{|V(G)|}}{{(|E(G)|p+p-1)!} \over {(p-1)}!(p!)^{|E(G)|}}e(\mathcal {P}(G,r)). \end{aligned}$$

Since $p> |V(G)|+|E(G)|$, $(|V(G)|p+p-1)!$ is divisible by $p^{|V(G)|}$, but not by $p^{|V(G)|+1}$ and $(|E(G)|p+p-1)!$ is divisible by $p^{|E(G)|}$, but not by $p^{|E(G)|+1}$. Therefore, it is readily seen that

$$\begin{aligned} \left( s!{{2p-1} \atopwithdelims (){p}}\right) ^r {{(|V(G)|p+p-1)!} \over {(p-1)}!(p!)^{|V(G)|}}{{(|E(G)|p+p-1)!} \over {(p-1)}!(p!)^{|E(G)|}} \end{aligned}$$

is not divisible by *p*. We will denote this latter expression as $c_p$; note that $c_p$ corresponds to the constant $A_p$ introduced at the beginning of this section. Hence, it is easy to see that if $\#EC (G,r)$ denotes the actual number of equitable coloring of *G* with *r* colors, then

$$\begin{aligned} e(\mathcal {P}(G,r,p))\equiv c_p e(\mathcal {P}(G,r)) \#EC (G,r)\mod p. \end{aligned}$$

Since $c_p$ is not divisible by *p*, it is clear that $e(\mathcal {P}(G,r,p))\not \equiv 0 \mod p$ if and only if $e(\mathcal {P}(G,r))\cdot \#EC (G,r)\not \equiv 0 \mod p$, and the claim holds. $\square$

### Claim 6

If $\#EC (G,r)\ne 0$, then there is a prime number *p* greater than $2r|V(G)|+r^2|E(G)|$ and smaller than $(2r|V(G)|+r^2|E(G)|)^2$ such that *p* does not divide $e(\mathcal {P}(G,r))\cdot \#EC (G,r)$.

### Proof

(of the Claim) Let us first upperbound $e(\mathcal {P}(G,r))\#EC (G,r)$. Clearly, $\mathcal {P}(G,r)$ contains $m = 2(r-1)|V(G)|+(r^2-1)|E(G)|$ elements, hence $e(\mathcal {P}(G,r))\le m!$. It can easily be verified that the number of possibilities of dividing $|V(G)|=rs$ vertices into *r* color classes with exactly *s* colors each is ${{(rs)!} \over {(s!)^r}}$. By Fact 1, the product of all primes between $2r|V(G)|+r^2|E(G)|$ and $(2r|V(G)|+r^2|E(G)|)^2$ is at least $(2r|V(G)|+r^2|E(G)|)!2^{2r|V(G)|+r^2|E(G)|}$. However, $2(r-1)|V(G)|+(r^2-1)|E(G)|+ |V(G)| \le 2r|V(G)|+r^2|E(G)|$ and hence $e(\mathcal {P}(G,r))\#EC (G,r)\le (2(r-1)|V(G)|+(r^2-1)|E(G)|)!+ {{|V(G)|!}\over {(s!)^r}}$ is clearly less than the product of all primes between $2r|V(G)|+r^2|E(G)|$ and $(2r|V(G)|+r^2|E(G)|)^2$. Note that if a natural number *N* is divisible by set of primes $p_1,\dots , p_\ell$ then *N* is divisible by the product of these primes and in particular *N* is bigger than the product of these primes. Therefore, $e(\mathcal {P}(G,r))\#EC (G,r)$ cannot be divisible by all primes between $2r|V(G)|+r^2|E(G)|$ and $(2r|V(G)|+r^2|E(G)|)^2$, from which the claim follows. $\square$

### Claim 7

$$tw (C(\mathcal {P}(G,r,p)))\le r\cdot (tw (G)+3)+6$$.

### Proof

(of the Claim) To distinguish vertices of *G* and $C(\mathcal {P}(G,r,p))$ in this proof, we will refer to the vertices of $C(\mathcal {P}(G,r,p))$ as elements. So, let $\mathcal {T}= (T, \{X_{t}\}_{t\in V(T)})$ be a nice tree-decomposition of *G* of width $tw (G)$. Using $\mathcal {T}$, we show how to construct a tree-decomposition $\mathcal {T}' = (T', \{X_{t}'\}_{t\in V(T')})$ of $C(\mathcal {P}(G,r,p))$ with treewidth at most $r\cdot (tw (G)+3)+6$. The construction can be summarized as follows:

1. All bags of $\mathcal {T}'$ will contain the anchors $a_0,\dots , a_{r+3}$ as well as the top-most element of each stalk of the (|*V*(*G*)|, *s*)-flowers in the first *r* levels; let $\delta$ denote this set of $2r+4$ elements.
2. For every bag $t\in \mathcal {T}$, the tree-decomposition $\mathcal {T}'$ will contain a node $t'$ such that if $v\in X_{t}$ then $\{v^1,\dots , v^r\}\in X_{t}'$.
3. Afterwards, every introduce node $t\in \mathcal {T}$ that introduces a vertex *v* will be replaced by a long path $P_t$ which gradually introduces and subsequently forgets all remaining elements associated with the flower of *v* at level $r+1$ (i.e., the stalk) as well as every petal from the first *r* levels associated with *v* .
4. For each edge *e*, we pick an introduce node $t\in \mathcal {T}$ which contains both endpoint of *e* and extend the path $P_t$ by a new segment which introduces and subsequently forgets all elements associated with the flower of *e* at level $r+2$.
5. The root node is replaced by a path that takes care of all elements which are not associated with any vertex or edge in *G*.

Let us now take a closer look what happens in $\mathcal {T}'$ when $t\in T$ is an introduce node. Let $v\in V(G)$ be the vertex introduced at *t*. Since $X_{t}'$ contains elements $a_0,\dots , a_{r+2}, v^1,\dots , v^r$, and the maximum element of each stalk of the (|*V*(*G*)|, *s*)-flower in the first *r* levels, it is easily seen that every petal from the first *r* levels associated with *v* as well as the stalk of the flower associated with *v* in level $L_{r+1}$ each forms a separate connected component of $C(\mathcal {P}(G,r,p)){\setminus } X_{t}'$. Moreover, for an edge $e=uv$ such that $X_t$ contains both endpoints of *e*, we have that $X_t'$ also contains elements $u^1,\dots , u^r$ and one can see that also the flower associated with *e* at level $L_{r+2}$ is a connected component of $C(\mathcal {P}(G,r,p)){\setminus } X_{t}'$. It is readily seen that the singleton, chain, and flower all have pathwidth 1 and hence there is a nice path-decomposition $(B_1, \dots , B_{\ell _t})$ with $B_1 = B_{\ell _t} = \emptyset$ of the graph containing every petal element from first *r* levels associated with *v*, the stalk of the flower associated with *v* in level $L_{r+1}$, and the flower associated with *e* in level $L_{r+2}$ for every edge *e* introduced at *t*. We then replace $X_{t}'$ by a path $(Y_1,\dots , Y_{\ell _t})$ such that $Y_i=B_i\cup X_{t}'$ and for each $i\in \{1,\dots ,\ell _t-1\}$ the node with bag $Y_{i+1}$ is the parent of the node with the bag $Y_i$.

It is easy to see now, that when we are forgetting vertex *v* in node *t* in $\mathcal {T}$, we can forget elements $v^1,\dots , v^r$ in $\mathcal {T}'$, because we already introduced all its adjacent edges in $C(\mathcal {P}(G,r,p))$ either in the path corresponding to the node of $\mathcal {T}$ introducing *v* or the one introducing a neighbor of *v*.

Finally, when we get to the root node of $\mathcal {T}$, we have already forgotten all elements associated with any specific vertex or edge of *G*. Therefore, the only elements besides $\delta$ which need to be included in $\mathcal {T}'$ are the remaining elements in the stalks in the first *r* levels and the sticks in every level. However, it is easy to see that at this point they all form separate chains in $C(\mathcal {P}(G,r,p)){\setminus } \delta$. Hence there once again exists a path-decomposition of width at most $|\delta |+2$ which gradually introduces and subsequently forgets all of these elements.

One can readily see that the properties (T1), (T2), and (T3) are satisfied and we are only left with computing the width of $\mathcal {T}'$. By construction, every join and forget node in $\mathcal {T}$ will become a node in $\mathcal {T}'$ whose bag has size at most $r\cdot |X_t|+2r+4$. On the other hand, every introduce node in $\mathcal {T}$ will become a path in $\mathcal {T}'$, and the largest bag on this path has size at most $r\cdot |X_t|+2r+6$, from which the claim follows. $\square$

*Concluding the proof* Let us summarize the fpt turing reduction used to prove Theorem 1. Given an instance (*G*, *r*) of Equitable Coloring[tw], we loop over all primes *p* such that $2r|V(G)|+r^2|E(G)|<p<(2r|V(G)|+r^2|E(G)|)^2$, and for each such prime we construct the poset $\mathcal {P}(G,r,p)$; from Claim 6 it follows that if $\#EC (G,r)\ne 0$, then at least one such prime will not divide $e(\mathcal {P}(G,r))\cdot \#EC (G,r)$, and by Claim 7 the cover graph of each of the constructed posets *P*(*G*, *r*, *p*) has bounded treewidth. For each such poset $\mathcal {P}(G,r,p)$, we compute $e(\mathcal {P}(G,r,p))$ by the black-box procedure provided as part of the reduction. If for any prime *p* we get $e(\mathcal {P}(G,r,p))\not \equiv 0\mod p$, then we conclude that (*G*, *r*) is a yes-instance, and otherwise we reject (*G*, *r*), and this is correct by Claim 5. $\square$

## Fixed-Parameter Tractability of Counting Linear Extensions

This section is dedicated to proving our algorithmic result, stated below.

### Theorem 2

#LE is fixed-parameter tractable parameterized by the treewidth of the incomparability graph of the input poset.

The proof of Theorem 2 is divided into two steps. First, we apply a transformation process to a path-decomposition $\mathcal {Q}$ of small width (the existence of which is guaranteed by Corollary 1) of $I(\mathcal {P})$ which results in a tree-decomposition $\mathcal {T}$ of $I(\mathcal {P})$ satisfying certain special properties. The properties of $\mathcal {T}$ are then used to prove that $I_C(\mathcal {P})$ has treewidth bounded by the treewidth of $I(\mathcal {P})$. In the second step, we construct an MSO formulation which enumerates all the linear extensions of $\mathcal {P}$ using $I_C(\mathcal {P})$, and apply Fact 4.

### The Treewidth of Combined Graphs

We begin by arguing a useful property of separators in incomparability graphs.

#### Lemma 3

Let $S\subseteq V(I(\mathcal {P}))$. Then for each pair of distinct connected components $C_1, C_2$ in $I(\mathcal {P}) -S$, it holds that for any $a_1,b_1\in C_1$ and any $a_2,b_2\in C_2$ we have $a_1\le a_2$ iff $b_1\le b_2$. Namely, the poset contains a total order of all connected components in $I(\mathcal {P}) -S$.

#### Proof

We begin by proving the following claim.

#### Claim 8

Let *a*, *b*, *c* be three distinct elements of $\mathcal {P}$ such that $a \parallel b$ and both pairs *a*, *c* and *b*, *c* are comparable. Then $a \le c$ iff $b \le c$.

#### Proof

(of the Claim) Suppose that, w.l.o.g., $a\le c$ and $c\le b$. Then by the transitivity of $\le$, we get $a\le b$ which contradicts our assumption that $a\parallel b$. $\square$

Now to prove Lemma 3, assume for a contradiction that, w.l.o.g., there exist $a_1,b_1\in C_1$ and $a_2, b_2\in C_2$ such that $a_1\le b_1$ and $b_2\le a_2$. Let $Q_1$ be an $a_1$-$a_2$ path in $I[C_1]$. By Claim 8, $a_1\le b_1$ implies that every element *q* on $Q_1$ satisfies $q\le b_1$, and in particular $a_2\le b_1$. Next, let $Q_2$ be a $b_1$-$b_2$ path in $I[C_2]$. Then Claim 8 also implies that each element $q'$ on $Q_2$ satisfies $a_2\le q'$. Since $b_2$ lies on $Q_2$, this would imply that $a_2\le b_2$, a contradiction. $\square$

To proceed further, we will need some additional notation. Let $\mathcal {T}=(T,\mathcal {X})$ be a rooted tree-decomposition and $t\in T$. We denote by *L*(*t*) the set of all vertices which occur in the “branch” of $T-t$ containing the root *r*; formally, $L(t)=\{v\in X_{t'}{\setminus } X_t ~ | ~ t'$ lies in the same connected component as $r \text { in }T-t\}$. We then set $R(t)=V(G){\setminus } (L(t)\cup X_t)$. We also let $T^r_t$ denote the connected component of $T-t$ which contains the root *r*.

Next, recall that each connected component of the graph obtained after deleting $X_t$ must lie in a subtree of $T-t$ (Fact 3). Let $\varUpsilon _t$ be the set of connected components of $(I(\mathcal {P}){\setminus } X_t)\cap R(t)$. Recall that because of Lemma 3, the components of $\varUpsilon _t$ are totally ordered by $\mathcal {P}$. We say that two components $B_1$, $B_2\in \varUpsilon _t$ are *consecutive* if there is no element $v\in V(I(\mathcal {P})){\setminus } (X_t\cup B_1\cup B_2)$ that is in between elements in these components; formally, for every *v* it holds that either $v\le b$ for all $b\in B_1\cup B_2$, or $v\ge b$ for all $b\in B_1\cup B_2$.

A *block* of a bag $X_t$ is a maximum set of consecutive connected components in $(I(\mathcal {P})-X_t)\cap R(t)$; note that each block has a natural total ordering among the contained components, given by Lemma 3. We say that a node $t\in T$ has *z* blocks if there exist *z* distinct blocks of $X_t$. Blocks will play an important role in the tree-decomposition we wish to obtain from our initial path-decomposition of $I(\mathcal {P})$. The following lemma captures the operation we will use to alter our path-decomposition.

#### Lemma 4

Let $\mathcal {T}=(T,\mathcal {X})$ be a rooted tree-decomposition of a graph *G* and let $t\in T$ be such that there are *z* blocks of $X_t$. Then there is a tree-decomposition $\mathcal {T}'(T',\mathcal {X}')$ satisfying:

1. The width of $\mathcal {T}'$ is at most the width of $\mathcal {T}$.
2. The tree $T'$ contains $T^r_t$ as a subtree which is separated from the rest of $T'$ by *t*.
3. The degree of *t* in $T'$ is $z+1$.
4. There exists a bijection $\alpha$ between the *z* blocks of $X_t$ and the *z* trees in $T'-t$ other than $T^r_t$ such that for each block *B* of $X_t$, we have $\bigcup _{s\in \alpha (B)}X'_s{\setminus } X_t = B$.
5. For each $t'\in N[t]{\setminus } T^r_t$, we have $X_{t'}=X_t$.

#### Proof

It will be useful to observe that $T - T^r_t$ is a subtree of *T* and in particular it is connected. Consider the following construction of $\mathcal {T}'$. First, we copy all nodes of $T^r_t\cup \{t\}$ (along with their bags) into $\mathcal {T}'$, thus ensuring that Property **2** holds. Second, for each block *B* of $X_t$ we make a copy $T^B$ of the tree $T- T^r_t$, and connect the node $t^B$ corresponding to *t* in $T{\setminus } T^r_t$ by an edge to the node *t* in $T'$. Moreover, for each node $s\in T{\setminus } T^r_t$ we set $X'_{s^B}=X_s \cap (B\cup X_t)$. It is easy to verify that all of the required properties are now satisfied, and it remains to show that $\mathcal {T}'$ is indeed a tree-decomposition.

We argue that $\mathcal {T}'$ satisfies all three properties of tree-decompositions. Property (T1) follows directly from fact that $\mathcal {T}$ was tree decomposition, and hence every vertex that does not occur in a bag in $T^r_t$ must occur in some bag $X_s$ for some node $s\in T{\setminus } T^r_t$; then this vertex either also occurs in $X_t$ or occurs in some block *B* and hence in $X'_{s^B}$. Property (T2) is also straightforward, since each vertex either does not occur in any block or in exactly one block, and in both cases monotonicity follows from the monotonicity of $\mathcal {T}$ and the construction. For the final Property (T3), we recall that there are no edges between the blocks of $X_t$; in particular every edge $e=ab$ in $I(\mathcal {P})[X_t\cup R(t)]$ is either contained in $X_t$, goes between a vertex of $X_t$ and a vertex of some block *B*, or is contained in some block *B*. In all three cases, it holds that if $a,b\in X_s$ for some $s\in T{\setminus } T^r_t$, then $e\in X_{s^B}$ for some block *B*. Therefore, $\mathcal {T}'$ is a tree-decomposition and the proof is complete. $\square$

We proceed by showing how Lemma 4 is applied to transform a given path-decomposition.

#### Lemma 5

Let $\mathcal {Q}$ be a nice path-decomposition of $I(\mathcal {P})$. Then there is a rooted tree-decomposition $\mathcal {T}=(T,\mathcal {X})$ of $I(\mathcal {P})$ with the following properties. $\mathcal {T}$ is rooted at a leaf *r* and $X_r=\emptyset$, the width of $\mathcal {T}$ is at most the width of $\mathcal {Q}$, and for any node $t\in T$ with $z>1$ blocks:

1. The degree of *t* in *T* is $z+1$.
2. There exists a bijection $\alpha$ between the *z* blocks of $X_t$ and the *z* trees in $T'-t$ other than $T^r_t$ such that for each block *B* of $X_t$, we have $\bigcup _{s\in \alpha (B)}X_s {\setminus } X_t= B$.
3. For $t'\in N(t)\cap T^r_t$ there exists a vertex *v* such that $X_{t'}=X_t{\setminus } \{v\}$, and furthermore $t'$ has degree at most 2 and 1 block.
4. For each pair of neighbors $t,t'\in T$, it holds that $|X_t{\setminus } X_{t'}|+|X_{t'}{\setminus } X_t|\le 1$.

#### Proof

Let us order the vertices of $I(\mathcal {P})$ in the order in which they were introduced in $\mathcal {Q}$. We set the first leaf of $\mathcal {Q}$ to be the root *r*, and observe that $X_r=\emptyset$ since $\mathcal {Q}$ is nice. We then process the vertices of $I(\mathcal {P})$ in their order of introduction; when processing each such vertex *v*, we apply Lemma 4 to the unique node *t* of the current tree-decomposition which is closest to *r* and contains *v*; with a slight abuse of terminology, we say that *t* is the node where *v* is introduced. We show that the following invariants hold after (and before) each step of this procedure:

1. For each pair of neighbors $t,t'\in T$, it holds that $|X_t{\setminus } X_{t'}|+|X_{t'}{\setminus } X_t|\le 1$.
2. For each vertex *u* that was already processed, the introduce node of *u* satisfies the conditions of the lemma.
3. Any node *t* of degree greater than 2 is an introduce node of an already processed vertex.

Clearly, all invariant conditions hold for $\mathcal {Q}$ rooted at *r* (the first invariant condition follows by the fact that $\mathcal {Q}$ is nice, and the remaining two are satisfied trivially). Similarly, all invariant conditions hold after applying Lemma 4 on the first introduce node *t* in $\mathcal {Q}$. Indeed, note that since $X_r=\emptyset$, we can assume w.l.o.g. that *t* is a child of *r* and $X_t=\{v\}$ for some vertex *v*. Then the first and third invariant condition is immediately seen to hold; as for invariant condition 2, if *t* has more than 1 block then applying Lemma 4 ensures that *t* satisfies Conditions **1**, **2**, and **4**, while Condition **3** holds since $t'=r$.

For the induction step, suppose that the conditions hold in a tree-decomposition $\mathcal {T}$ obtained by inductively applying Lemma 4 as above, and the first unprocessed vertex is *v*. Let *t* be the unique node where *v* is introduced, and let $\mathcal {T}'$ be the tree-decomposition we obtained by applying Lemma 4 on $\mathcal {T}$ and *t*.

It is easy to verify that $\mathcal {T}'$ then satisfies the desired Conditions **1**, **2**, and **4** at the node *t* by Lemma 4. As for Condition **3**, since *t* is the introduce node of *v* and the first invariant condition holds in $\mathcal {T}$, it is clear that for $t'\in N(t)\cap T^r_t$ it is the case that $X_{t'}=X_t{\setminus } \{v\}$. Moreover, $t'$ cannot be an introduce node, since then $t'$ would have to introduce an already processed vertex, which would imply that $X_{t'} = X_{t}$ due to our application of Lemma 4 on introduce nodes. So, let us consider the node *s* on the unique $t'$-*r* path that is the closest introduce node to $t'$, and let $s'$ be the neighbor of *s* on the *s*-$t'$ path. Since no vertex was introduced on the $s'$-$t'$ path, it follows that $R(s')=R(t')$. Since $s'$ only has 1 block by the construction, it must be the case that $t'$ also only has one block, and so Condition **3** also holds.

We proceed by arguing that the invariant conditions remain satisfied by $\mathcal {T}'$. Since $\mathcal {Q}$ was nice and $\mathcal {T}$ satisfied the first invariant condition, it is readily seen that the first invariant condition holds for all pairs of neighbors in $T^r_t$ as well as for *t* with all of its neighbors. If $s^B$ and $s'^B$ are neighbors in a tree $\alpha (B)$ of $T'-t$, then by the construction in Lemma 4 there exists a pair of neighbors $s,s'\in T{\setminus } T^r_t$ such that $X'_{s^B}=X_s \cap (B\cup X_t)$ and $X'_{s'^B}=X_{s'} \cap (B\cup X_t)$. But then $|X'_{s^B}{\setminus } X'_{s'^B}|+|X'_{s'^B}{\setminus } X'_{s^B}| = |(X_s \cap (B\cup X_t)){\setminus } (X_{s'} \cap (B\cup X_t))|+|(X_{s'} \cap (B\cup X_t)){\setminus } (X_s \cap (B\cup X_t))| \le |X_{s}{\setminus } X_{s'}|+|X_{s'}{\setminus } X_{s}|\le 1$ and the first invariant condition follows. As for the second invariant condition, notice that from the construction it follows that all the vertices that precede *v* in the order of introduction in $\mathcal {Q}$ must have been introduced in some node of $T^r_t$, and the application of Lemma 4 does not alter such introduce nodes for previously processed vertices. Finally, by the induction hypothesis all nodes of $T{\setminus } T^r_t$ have degree at most 2, therefore from the construction in Lemma 4 it is clear that all nodes in $T'{\setminus } (T^r_t\cup \{t\})$ also have degree 2. Since all introduce nodes of unprocessed vertices lie in $T'{\setminus } (T^r_t\cup \{t\})$, we conclude that the third invariant condition also holds in $\mathcal {T}'$.

Now, let us consider the tree-decomposition $\mathcal {T}$ obtained after processing all vertices of $I(\mathcal {P})$ according to the procedure described above. $\mathcal {T}$ satisfies Condition **4** due to the first invariant of our procedure, and for all other conditions it suffices to consider nodes with more than 1 block. In particular, it suffices to verify that all such nodes satisfy the conditions of Lemma 4 and additionally also condition **3** of this Lemma. So, suppose for a contradiction that there exists a node *t* which does not meet these conditions, but all nodes on the unique *t*-*r* path do. Then there are two possibilities to consider for the unique neighbor $t'$ of *t* on the *t*-*r* path. If $t'$ were to have more than 1 block, then by our assumptions $t'$ would have to satisfy the conditions of Lemma 4, contradicting the fact that *t* has more than 1 block. On the other hand, if $t'$ were to have only a single block, then by construction *t* must be an introduce node of some vertex *v* and by our invariants and the construction it follows that *t* in fact must satisfy all the required conditions. In particular we conclude that all nodes *t* satisfy Conditions **1-4** and the lemma follows. $\square$

We call a tree-decomposition rooted at a leaf with $X_r=\emptyset$ which satisfies the properties of Lemma 5 a *blocked tree-decomposition*. The next ingredient we will need for proving that $I_C(\mathcal {P})$ has small treewidth is the notion of *cover-guards*.

Let $\mathcal {T}=(T,\mathcal {X})$ be a tree-decomposition of $I(\mathcal {P})$ rooted at *r* and let $t\ne r$. Then the *cover-guard* of *t*, denoted $\mathcal {A}_t$, is the set of vertices in *L*(*t*) which are incident to a cover edge whose other endpoint lies in *R*(*t*); formally, $\mathcal {A}_t=\{v\in L(t) ~|~ \exists u\in R(t): \{uv\}\in E(C(\mathcal {P})$. For a vertex $v\in I(\mathcal {P})$, we let $\mathcal {A}^v=\{t\in T~|~v\in \mathcal {A}_t\}$ and $X^v=\{t\in T~|~v\in X_t\}$.

Our next aim is to add all the cover-guards into each bag. Below, we show that this does not increase the size of bags too much.

#### Lemma 6

Let $\mathcal {T}=(T,\mathcal {X})$ be a blocked tree-decomposition of $I(\mathcal {P})$ of width *k*. Then for each $t\in T$ it holds that $|\mathcal {A}_t|\le 2 k+2$.

#### Proof

First, observe that if a node $t\in T$ has 0 blocks, then $R(t)=\mathcal {A}_t=\emptyset$. So, consider a node *t* which has exactly 1 block consisting of connected components $(D_1,\dots ,D_j)$ in $(I(\mathcal {P})-X_t)\cap R(t)$.

#### Claim 9

$$|\mathcal {A}_t|\le 2k+2$$.

#### Proof

(of the Claim) Assume for a contradiction that $|\mathcal {A}_t|>2k+2$. By Lemma 3 we have that $(D_1,\dots ,D_j)$ are consecutive connected components in a total order of connected components in $I(\mathcal {P})-X_t$. Hence any edge in $C(\mathcal {P})-X_t$ between *R*(*t*) and *L*(*t*) must necessarily have one endpoint in $D_1 \cup D_j$. Furthermore, an element in $\mathcal {A}_t$ cannot be adjacent to both $D_1$ and $D_j$ in $C(\mathcal {P})-X_t$ due to transitivity and acyclicity. So, we may partition $\mathcal {A}_t$ into $\mathcal {A}_t^1=\{v\in \mathcal {A}_t~|~\exists u\in D_1: v\lhd ^\mathcal {P}u\}$ and $\mathcal {A}_t^2=\{v\in \mathcal {A}_t~|~\exists u\in D_j: u\lhd ^\mathcal {P}v\}$.

By Lemma 3, it also follows that $\mathcal {A}_t^1$ and $\mathcal {A}_t^2$ must each lie in separate connected components of $I(\mathcal {P})-X_t$, say $C^1$ and $C^2$ respectively. Furthermore, each element in $\mathcal {A}_t^1$ is maximal in $C^1$ and each element in $\mathcal {A}_t^2$ is minimal in $C^2$. In particular, each of $\mathcal {A}_t^1$, $\mathcal {A}_t^2$ forms a clique in $I(\mathcal {P})$. But by our assumption on the size of $\mathcal {A}_t$, at least one of $\mathcal {A}_t^2$ and $\mathcal {A}_t^1$ must have size greater than $k+1$, which implies that $I(\mathcal {P})$ contains a clique of size at least $k+2$. It is well-known that each clique must be completely contained in at least one bag of a tree-decomposition, and so we arrive at a contradiction with $tw (I(\mathcal {P}))\le k$. Hence we conclude that $|\mathcal {A}_t|\le 2k+2$ and the claim holds. $\square$

Finally, consider a node *t* which has at least 2 blocks. By Property **3** of Lemma 5, it holds that *t* has a neighbor $t'$ in $T^r_t$ such that $X_{t'}=X_t{\setminus } \{v\}$ and $t'$ has 1 block. By Claim 9 we know that $\mathcal {A}_{t'}\le 2k+2$. Since $L(t)=L(t')$ and $R(t)\subseteq R(t')$, it follows that $\mathcal {A}_t\subseteq \mathcal {A}_{t'}$, and in particular $|\mathcal {A}_t|\le |\mathcal {A}_{t'}|$. We have now proved the desired bound for all nodes in $\mathcal {T}$, and so the lemma holds. $\square$

The following lemma allows us to argue that adding cover-guards into each bag still results in a tree-decomposition; it is worth noting that the assumption that the decomposition is blocked is essential for the lemma to hold.

#### Lemma 7

Let $\mathcal {T}=(T,\mathcal {X})$ be a blocked tree-decomposition of $I(\mathcal {P})$ rooted at *r* and let $v\in I(\mathcal {P})$. Then $T[\mathcal {A}^v\cup X^v]$ is a tree.

#### Proof

Since $\mathcal {T}$ is a tree-decomposition, we have that $T[X^v]$ must be a tree. Consider a connected component *A* of $T[\mathcal {A}^v]$ and its unique *A*-$X^v$ path *Q*, with endpoints $x\in X^v$ and $a\in A$. Since *r* is located at a leaf of *T* it must hold that $r\not \in Q$. We consider two cases: either *r* lies in the same connected component as $X^v$ in $T-Q$, or it lies in a different connected component.

In the former case, it follows that each internal vertex *q* of *Q* satisfies $R(a)\subseteq R(q)$ and $v\in L(q)$. But then by the definition of $\mathcal {A}^v$ and the fact that $a\in \mathcal {A}^v$, this would imply $q\in \mathcal {A}^v$, contradicting our construction of *Q*. Hence if *r* lies in the same connected component as $X^v$ in $T-Q$, then *A* is adjacent to $X^v$.

In the latter case, there must exist a node $q\in Q$ of degree at least 3 such that each of *A*, $X^v$ and *r* occur in different components of $T-q$. By the definition of $\mathcal {A}^v$, there exists a vertex $u\in R(a)$ such that $v\lhd ^\mathcal {P}u$ or $u\lhd ^\mathcal {P}v$. Since $u,v\in R(q)$ due to the location of the root and there is a cover edge between them, it follows that either *u*, *v* occur in the same connected component of $X_q$ or in two consecutive ones, but in either case *u*, *v* must lie in the same block of *q*, say block *B*. But since $u,v\not \in X_q$, this contradicts Property **2** in Lemma 5; indeed, each tree in $T-q$ contains at most one of *v*, *u* in its bags, and hence there exists no tree $T'$ in $T-q$ satisfying $\bigcup _{t'\in T'}X_{t'} {\setminus } X_q= B$. Hence *r* cannot occur in a different connected component than $X^v$ in $T-Q$.

We conclude that *Q* contains no internal vertices. In particular, every connected component of $T[\mathcal {A}^v]$ is adjacent to $X^v$. $\square$

With Lemmas 6 and 7, we have the tools necessary for arguing that there exists a tree-decomposition of the combined graph of small width.

#### Lemma 8

Let $\mathcal {T}=(T,\mathcal {X})$ be a blocked tree-decomposition of $I(\mathcal {P})$ such that $tw (\mathcal {T})\le k$. Then there exists a tree-decomposition $\mathcal {T}'$ of $I_C(\mathcal {P})$ of width at most $3k+2$.

#### Proof

Consider the tree-decomposition $\mathcal {T}'=(T,\mathcal {X}')$ where $\mathcal {X}'=\{X'_t~|~t\in T\}$ is defined as follows. For each $t\in T$ such that its unique neighbor *s* in $T^r_t$ satisfies $|X_{t}{\setminus } X_{s}|=1$, we set $X'_t=X_t\cup \mathcal {A}_s$; it will be useful to observe that $\mathcal {A}_s \supseteq \mathcal {A}_t$. For all other nodes $t\in T$, we then set $X'_t=X_t\cup \mathcal {A}_t$. We call nodes of the first type *non-standard* and nodes of the second type standard.

First, we note that the size of each bag in $\mathcal {T}'$ is at most $3k+2$, since every node $t\in T$ satisfies $|\mathcal {A}_t|\le 2k+2$ by Lemma 6. Furthermore, $\mathcal {T}'$ satisfies condition (T1) because $\mathcal {T}$ was a tree-decomposition of $I(\mathcal {P})$. $\mathcal {T}'$ also satisfies condition (T2); indeed, for each $v\in \mathcal {P}$ it holds that $X'^v$ restricted to standard nodes is a connected tree by Lemma 7, and by construction every non-standard node *t* such that $v\in X'_t{\setminus } X_t$ is adjacent to a standard node containing *v*. So, it only remains to argue condition (T3).

Obviously, condition (T3) holds for any edge of $I(\mathcal {P})$. So, consider two elements *u*, *v* of $\mathcal {P}$ such that $u\lhd ^\mathcal {P}v$ or $v\lhd ^\mathcal {P}u$. If there exists a node $t\in T$ such that $u,v\in X_t$, then $u,v\in X'_{t}$ and the condition also holds for this edge in $I_C(\mathcal {P})$. So, assume that $X^v$ and $X^u$ are disjoint and let *Q* be the unique $X^v$-$X^u$ path in *T*. By Property **4**, the $X^v$-$X^u$ path *Q* in *T* must contain at least one internal node.

Consider the case where one of these subtrees, say w.l.o.g. $X^v$, lies in the connected component $T^r_t$ of $T-Q$. Then for each internal node $q\in Q$, it holds that $v\in L(q)$ and $u\in R(q)$, which in turn implies that $v\in \mathcal {A}_q$. Let $q_u$ be the endpoint of *Q* in $X^u$ and let $q_0$ be the neighbor of $q_u$ in *Q*. By Property **4** we have $X_{q_u}{\setminus } X_{q_0}=\{u\}$, which implies that $q_u$ is a non-standard node and in particular $\mathcal {A}_{q_0}\subseteq X'_{q_u}$. Since $q_0$ is an internal node of *Q*, it follows that $v\in X'_{q_u}$ which means that condition (T3) also holds for any edge *uv* in this case.

Finally, consider the case where there exists a node $q\in Q$ of degree at least 3 such that each of $X^u$, $X^v$ and *r* occur in different components of $T-q$. Then we reach a contradiction similarly as in the proof of Lemma 7. In particular, since $u,v\in R(q)$ due to the location of the root and there is a cover edge between them, it follows that either *u*, *v* occur in the same connected component of $X_q$ or in two consecutive ones, but in either case *u*, *v* must lie in the same block of *q*, say block *B*. But since $u,v\not \in X_q$, this contradicts Property **2** in Lemma 5; indeed, each tree in $T-q$ contains at most one of *v*, *u* in its bags, and hence there exists no tree $T'$ in $T-q$ satisfying $\bigcup _{t'\in T'}X_{t'} {\setminus } X_q= B$. Hence this case in fact violates our assumptions and cannot occur.

Summarizing the above arguments, we conclude that each bag in $\mathcal {T}'$ has size at most $3k+2$ and that $\mathcal {T}'$ satisfies all of the conditions of a tree-decomposition. $\square$

#### Corollary 2

Let $\mathcal {P}$ be a poset such that $tw (I(\mathcal {P}))\le k$. Then $tw (I_C(\mathcal {P}))\le 3k+2$.

#### Proof

By Corollary 1 we know that there exists a nice path-decomposition of $I(\mathcal {P})$ of width at most *k*. By Lemma 5, it follows that there exists a blocked tree-decomposition of $I(\mathcal {P})$ of width at most *k*. The corollary then follows by Lemma 8. $\square$

### MSO Formulation

In this subsection, we use Fact 4 to prove the following result, which forms the second ingredient required for our proof of Theorem 2.

#### Lemma 9

#LE is fixed-parameter tractable parameterized by the treewidth of the combined graph of the input poset.

#### Proof

Let $\mathcal {P}:=(P,\le ^P)$ be a poset. Let *G* be an (edge-)labeled directed graph obtained from $I_C(\mathcal {P})$ by directing every bidirectional edge of $I_C(\mathcal {P})$, i.e., every edge of $I(\mathcal {P})$, in an arbitrary way and labeling it with the label $\parallel$.

For a set of edges $E \subseteq E(G)$ with label $\parallel$, let *G*[*E*] be the graph obtained from *G* after reversing every edge in *E*. Moreover, for a linear extension $\preceq$ of $\mathcal {P}$ let $E_G(\preceq )$ be the set of edges (*u*, *v*) of *G* such that $v \preceq u$. Note that because every linear extension of $\mathcal {P}$ has to respect the direction of the edges in *G* given by $C$, it holds that every edge in $E_G(\preceq )$ has label $\parallel$.

#### Claim 10

$E_G(\preceq )$ defines a bijection between the set of linear extensions of $\mathcal {P}$ and the set of subsets *E* of edges of *G* with label $\parallel$ such that *G*[*E*] is acyclic.

#### Proof

(of the Claim) Let $\preceq$ be a linear extension of $\mathcal {P}$. Then, as observed above, $E_G(\preceq )$ is a set of edges of *G* with label $\parallel$. Moreover, because $G[E_G(\preceq )]$ is a subgraph of $P_G(\preceq )$ and $P_G(\preceq )$ is acyclic so is $G[E_G(\preceq )]$. Hence, $E_G(\preceq )$ is a function from the set of linear extensions of $\mathcal {P}$ to the set of subsets *E* of edges of *G* with label $\parallel$ such that *G*[*E*] is acyclic. Towards showing that $E_G(\preceq )$ is injective, assume for a contradiction that this is not the case, i.e., there are two distinct linear extensions $\preceq _1$ and $\preceq _2$ of $\mathcal {P}$ such that $E_G(\preceq _1)=E_G(\preceq _2)$ and let *u* and *v* be two elements of $\mathcal {P}$ ordered differently by $\preceq _1$ and $\preceq _2$. Then $\{u,v\} \in I(\mathcal {P})$ and hence either $(u,v) \in G$ or $(v,u) \in G$ the label of (*u*, *v*) or (*v*, *u*) respectively is $\parallel$. W.l.o.g. assume that $(u,v) \in G$ with label $\parallel$. But then, because $\preceq _1$ and $\preceq _2$ differ on *u* and *v*, either $(u,v) \in E_G(\preceq _1)$ but not $(u,v) \in E_G(\preceq _2)$ or $(u,v) \in E_G(\preceq _2)$ but not $(u,v) \in E_G(\preceq _1)$. In both cases we get a contradiction to our assumption that $E_G(\preceq _1)=E_G(\preceq _2)$.

It remains to show that $E_G(\preceq )$ is surjective. To see this let *E* be a subsets of the edges of *G* with label $\parallel$ such that *G*[*E*] is acyclic. Because *G*[*E*] is acyclic it has a topological ordering, say $\preceq$, of its vertices. Because *G*[*E*] contains $C(\mathcal {P})$ as a subgraph and any topological ordering of $C(\mathcal {P})$ is a linear extension of $\mathcal {P}$, we obtain that $\preceq$ is a linear extension and also $E=E_G(\preceq )$. $\square$

It follows from the above that instead of counting the number of linear extensions of $\mathcal {P}$ directly, we can count the number of subsets *E* of the edges of *G* with label $\parallel$ such that *G*[*E*] is acyclic. We will show next that there is an MSO formula $\varPhi (X)$, whose length is independent of *G* and can hence be considered constant, such that $G \models \varPhi (X)$ if and only if *X* is a subset of the edges of *G* with label $\parallel$ such that *G*[*E*] is acyclic. Because of Fact 4, this implies that #LE is fixed-parameter tractable when parameterized by $tw (G)$ and hence also when parameterized by $tw (I_C(\mathcal {P}))$, concluding the proof of the lemma.

Informally, $\varPhi (X)$ will check that *X* is a set of edges of *G* with label $\parallel$ and there is no non-empty set of edges *C* of *G*[*X*] that forms a cycle. For the definition of $\varPhi (X)$ we will need the following auxiliary formulas.

- The formula $edgesin (X):=\forall x X x \rightarrow P_\parallel x$, which holds if and only if *X* is a set of edges of *G* with label $\parallel$.
- The formula $edgesne (C):=\exists c C c \wedge (\forall c C c \rightarrow E c)$, which holds if and only if *C* is a non-empty set of edges of *G*.
- The formula $cyclic (C,X):=\forall v V v \rightarrow degree (C,X,v)$, which holds if and only if the set *C* of edges of *G*[*X*] is a disjoint union of directed cycles. Note that this implies that either *C* is empty or *C* contains at least one (directed) cycle.
- The formula $degree (C,X,v):= degree _0(C,X,v) \vee degree _2(C,X,v)$, which holds if and only if either no edge in *C* is incident to *v* or there are exactly two edges in *C* that are incident to *v* such that one of them corresponds to an edge in *G*[*X*] with tail *v* and the other corresponds to an edge in *G*[*X*] with head *v*.
- The formula $degree _0(C,X,v):=\lnot \exists c C c \wedge I vc$, which holds if and only if no edge in *C* is incident to *v*.
- The formula
  $$\begin{aligned} degree _2(C,X,v) :=&\,\, \exists c_i \exists c_o C c_i \wedge C c_o \wedge in (X,c_i,v) \wedge out (X,c_o,v) \wedge \\&\left( \forall c (C c \wedge \lnot c=c_i \wedge \lnot c=c_o) \rightarrow \lnot (H vc \vee T vc)\right) \\ \end{aligned}$$
  which holds if and only if there are exactly two edges in *C* that are incident to *v* such that one of them corresponds to an edge in *G*[*E*] with tail *v* and the other corresponds to an edge in *G*[*E*] with head *v*.
- The formula
  $$\begin{aligned} in (X,c,v)&:= (\lnot P_\parallel c \wedge H vc) \vee (P_\parallel c \wedge \lnot X c \wedge H vc) \vee (P_\parallel c \wedge X c \wedge T vc) \end{aligned}$$
  which holds if and only if *v* is the head of the edge of *G*[*X*] represented by *c*.
- The formula
  $$\begin{aligned} out (X,c,v)&:= (\lnot P_\parallel c \wedge T vc) \vee (P_\parallel c \wedge \lnot X c \wedge T vc) \vee (P_\parallel c \wedge X c \wedge H vc) \end{aligned}$$
  which holds if and only if *v* is the tail of the edge of *G*[*X*] represented by *c*.

Then $\varPhi (X)$ is the formula:

$$\begin{aligned} \varPhi (X) := edgesin (X) \wedge \lnot \left( \exists C edgesne (C) \wedge cyclic (C,X)\right) \end{aligned}$$

Which concludes the proof. $\square$

For completeness, we conclude the section by stating the proof of Theorem 2.

#### Proof

(of Theorem 2) Let $\mathcal {P}$ be the input poset and let $k=tw (I(\mathcal {P}))$. Then $tw (I_C(\mathcal {P}))\le 3k+2$ by Corollary 2, and the theorem follows by Lemma 9. $\square$

## Conclusions and Future Work

We have given the first parameterized intractability result for counting linear extensions. We hope that the employed techniques will inspire similar results and expand our knowledge about the parameterized complexity of counting problems. In particular, even for #LE there remain many open questions concerning other very natural parameterizations such as the width of the poset (which is, in fact, upper-bounded by the treewidth of the incomparability graph) or the treewidth of the poset graph. Moreover, our intractability result for the treewidth of the cover graph poses the question whether there are stronger parameterizations under which #LE becomes tractable, e.g., the treewidth of the comparability graph (i.e., the undirected graph underlying the poset graph), the treedepth or even vertex cover number of the comparability or cover graph, as well as combinations of these parameters with parameters such as the width, the dimension, or the height of the poset. These numerous examples illustrate that the parameterized complexity of #LE is still largely unexplored. As a side note it would also be interesting to establish whether our hardness result for #LE can be sharpened to $\#$W[1]-hardness and to obtain matching membership results.
